# Illuminating the life of GPCRs

**DOI:** 10.1186/1478-811X-7-16

**Published:** 2009-07-14

**Authors:** Ilka Böhme, Annette G Beck-Sickinger

**Affiliations:** 1 Institute of Biochemistry, Faculty of Biosciences, Pharmacy and Psychology, Leipzig University, Brüderstr. 34, 04103 Leipzig, Germany

## Abstract

The investigation of biological systems highly depends on the possibilities that allow scientists to visualize and quantify biomolecules and their related activities in real-time and non-invasively. G-protein coupled receptors represent a family of very dynamic and highly regulated transmembrane proteins that are involved in various important physiological processes. Since their localization is not confined to the cell surface they have been a very attractive "moving target" and the understanding of their intracellular pathways as well as the identified protein-protein-interactions has had implications for therapeutic interventions. Recent and ongoing advances in both the establishment of a variety of labeling methods and the improvement of measuring and analyzing instrumentation, have made fluorescence techniques to an indispensable tool for GPCR imaging. The illumination of their complex life cycle, which includes receptor biosynthesis, membrane targeting, ligand binding, signaling, internalization, recycling and degradation, will provide new insights into the relationship between spatial receptor distribution and function. This review covers the existing technologies to track GPCRs in living cells. Fluorescent ligands, antibodies, auto-fluorescent proteins as well as the evolving technologies for chemical labeling with peptide- and protein-tags are described and their major applications concerning the GPCR life cycle are presented.

## Review

### Introduction

G-protein coupled receptors (GPCRs) are integral membrane proteins, consisting of a single polypeptide chain with seven transmembrane domains (TMD). They control and influence a diversity of physiological functions by mediating the signal of a wide variety of stimuli such as peptide hormones, neurotransmitters, neuropeptides, autocrine factors and even photons. Thereby the ligand transmits its activity to an intracellular signal through activation of a heterotrimeric guanosine triphosphate-binding protein (G-protein) by the receptor. As a result, a broad range of downstream intracellular signals are activated, leading to both short-term effects (e.g. changes in intracellular calcium levels) and long-term effects (e.g. gene transcription). Representing the largest family of transmembrane signaling molecules in the human genome, GPCRs are a very important class of therapeutic targets for the pharmaceutical industry and nearly half of the drugs currently in use act on these biomolecules.

In addition to the binding of ligands and G-proteins, GPCRs interact with a broad range of other proteins with potential roles specifically in receptor biosynthesis, distribution, signaling, desensitization, clustering, internalization, trafficking and degradation. These include other GPCRs, GPCR kinases (GRKs), second-messenger-dependent kinases, arrestin molecules, molecular chaperones, receptor-activity-modifying proteins (RAMPs) and PDZ-domain-containing proteins [1]. For an excellent review that summarizes these activities including schematic figures see [2]. The relationship between agonist-induced activation of receptors, receptor translocation and cell function has previously been shown to be difficult to investigate because it is a dynamic process and localization of receptors by standard biochemical methods offers insufficient high-resolution spatial information. In addition, the expression levels of GPCRs are generally low in native systems, which make the detection even more difficult. But elucidating these interactions will help to understand their cellular functions in order to develop new and improved pharmaceuticals. Since there is evidence that several peptide hormone receptors are over-expressed in various human cancer cells it has been a challenge to develop regulatory, receptor-binding peptides as agents for tumor diagnosis and therapy. However receptor-mediated internalization is a prerequisite for this type of study [3]. Therefore novel methods to study receptor localization and function are needed as well as the extension of techniques to visualize and quantify involved biomolecules and processes with a spatiotemporal high-resolution and sensitivity [4].

Classically, receptors have been studied using radioactive isotopes, enzyme-linked immunosorbent assays (ELISAs) or functional responses in isolated tissue or organ preparations. The disadvantages of these methods, such as radioactive hazards and the limitations of studying the molecular dynamics of receptor activation have hindered advancements in receptor research. Biochemical methods for the investigation of protein-protein-interactions, such as co-immunoprecipitation assays, Western-blot analysis, "pulldown" approaches or yeast two-hybrid experiments have several drawbacks, e.g. artifacts owing to harsh techniques that are required to isolate membrane proteins, failure to identify components of a protein complex or false-positive as well as false-negative results. Therefore, non-invasive, real-time imaging methods applied to living cells have become very important in cell biology.

Fluorescence techniques that allow imaging of reporter gene expression, protein trafficking and monitoring of many dynamic biochemical signals have become feasible through the development of novel fluorophores as well as through the improvement of fluorescence instrumentation and advanced data analysis methods [5]. They are considered superior over other existing molecular detection technologies because of their enhanced sensitivity, minimal perturbation, multiplicity of measurable parameters and suitable time scales. This allows the analysis of several biologically relevant molecular processes [6]. Fluorescence is the most sensitive spectroscopic method. Reproducible signals from samples containing less than 1 nM concentrations of some fluorophores can be quantified. The signal can be analyzed by different methods, including its intensity, lifetime, energy (wavelength) and rotational freedom (polarization or anisotropy), to reveal different aspects of a structure, interaction, mechanism or process [7,8]. Furthermore, fluorescence is a non-destructive phenomenon, so any signal change can be monitored as a function of time to determine its kinetics. Of course it has to be considered that some of the fluorophores might be toxic in certain systems, either themselves or by generating free radicals.

Techniques such as scanning confocal microscopy (SCM) and fluorescence correlation spectroscopy (FCS) have offered the establishment of assays at the single cell [9] and the single molecule level [10]. Confocal and multiphoton microscopes coupled with sophisticated image analysis software packages are becoming affordable. The development of very high resolution, high sensitivity cameras and 3D deconvolution methods advances the area of quantitative 4D imaging [11]. Microscopy methods such as total internal reflection fluorescence microscopy (TIRFM) and interference reflection microscopy (IRM) can be used for selective imaging of the plasma membrane of cells, e.g. to study exo- and endocytic pathways or plasma membrane dynamics during internalization [12-14].

Fluorescent labeling reagents are an essential component of a huge industry built on sensitive fluorescence detection and reagents with close to maximum theoretical brightness are available in many different colors. Hundreds of small organic dyes for covalent labeling of macromolecules have been developed and industrially optimized in their wavelength range, brightness, photo-stability and reduced in self-quenching. Strategies have included extension of double-bond conjugation, rigidification through extra rings and decoration with electron-withdrawing or obligatorily charged substituents such as fluorines or sulfonates [15,16]. Labeling of proteins with fluorescent probes or affinity reagents has facilitated *in vitro *studies of protein structure, dynamics and protein-protein-interactions. However, traditional methods of protein labeling are often inadequate for *in vivo *studies, because they require purification of the protein, chemical labeling, re-purification and re-introduction into cells by invasive methods such as micro-injection or electroporation. These limitations have spawned efforts to label proteins in living cells or tissues non-invasively.

Appropriate methods for the study of receptor trafficking and regulation in native systems have not been available up to now. The limited axial (*z*) resolution of fluorescence and confocal-based microscopy impedes the imaging of receptors in individual cells deep within living tissues. The recent development and availability of fluorescent antibodies, fluorescent ligands and recombinant DNA technologies to label GPCRs in living cells provide new insights into their "real life" and "fate". Studies with GPCRs in living dissociated hepatocytes and vascular smooth muscle cells revealed quantitative data on receptor localization and translocation, which highly correlated with results obtained with fluorescent ligands or heterologous expression systems [9].

Subcellular distribution patterns have become an essential component of GPCR characterization that might have multiple regulatory consequences. For example, intracellular receptor pools that are rapidly transported to the cell membrane upon activation have been suggested to reduce desensitization and/or potentiate signaling. Many receptor subtypes have been shown to differ in their subcellular localization within the same cell type and particular receptors might localize differently depending on the cell type in which they are expressed. Unraveling these trafficking pathways and heterologous interactions by live imaging methods is strongly supported by the existence of various markers for intracellular compartments and pathways, as well as by inclusion of inhibitors for these processes, to fully understand the complex network [17].

**Figure 1:**
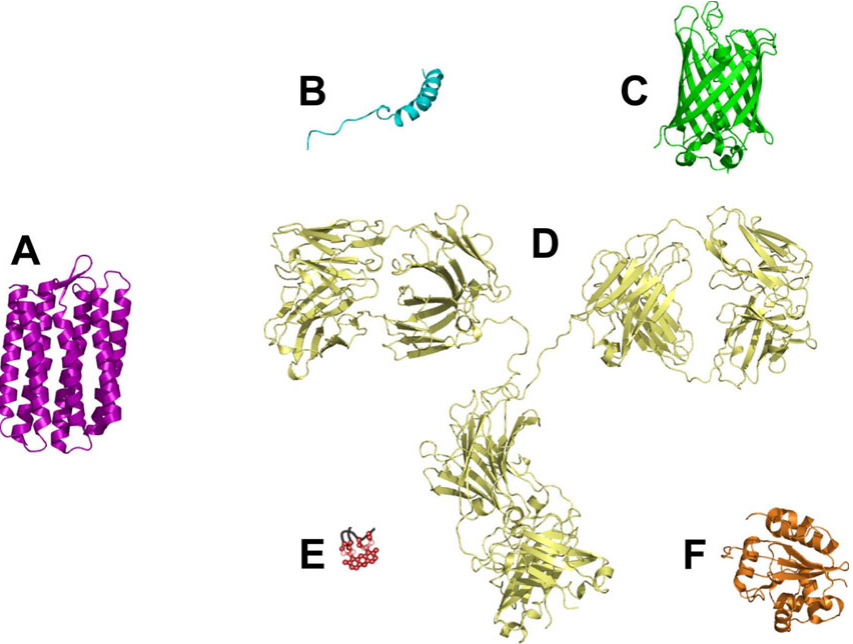
**Existing technologies to track GPCRs in living cells, represented at the same scale**. Representation of a GPCR based on the structure of sensory rhodopsin II (A; Protein Data Bank identifier (PDB ID), 1GUE), the structure of the peptide ligand porcine neuropeptide Y (B; PDB ID, 1F8P), the structure of an auto-fluorescent protein, namely the S65A/Y66F GFP variant (C; PDB ID, 2HGD), the structure of an immunoglobulin gamma (D; PDB ID 1IGT), the Lumio™-tag (E), the SNAP™-tag based on the structure of the human O^6^-alkylguanine-DNA alkyltransferase, (F; PDB ID, 1EH6).

This review will focus on the existing technologies to track GPCRs in living cells, such as fluorescent ligands and antibodies, auto-fluorescent proteins (AFPs) as well as peptide- and protein-tag technologies, such as the Lumio™- or SNAP™-tag (Figure 1). We aim to cover the major applications of these labeling methods in fluorescence imaging in order to provide a survey on the current state-of-the-art.

### Methods to label and visualize GPCRs

#### Fluorescent antibodies

Immunohistochemistry (IHC) is based on an antigen-antibody reaction. In the case of GPCR labeling the antigen is the receptor protein or a certain epitope tag and the antibody is a glycoprotein targeting a particular recognition sequence. In most cases the protein of interest (POI) is labeled with a primary antibody followed by amplification with a secondary antibody that is conjugated to a small organic dye or an enzyme. Alternatively, primary antibodies can also be directly conjugated to fluorophores or enzymes [18,19]. This is especially useful when antibodies are injected into living cells or when the increase of spectral diversity is required to analyze multiple proteins.

The availability of specific and potent antibody reagents is essential to obtain reliable and interpretable results in IHC studies. Accordingly, it has to be paid attention whether antibodies recognize the naturally folded protein as well. Antibodies against receptors can be generated in animals, e.g. rabbits, chickens or mice, through immunization with cells bearing receptors at their surface, injection of affinity-purified receptor or immunization with synthetic peptides derived from the nucleotide sequence of receptor genes [20]. In contrast to polyclonal antibodies (PAbs), monoclonal antibodies (MAbs) are directed against a single epitope of an antigen, which makes them extremely selective. The established phage display technology provides a more effective tool for their generation compared to hybridoma technology or immunization [21].

Receptor cloning and recombinant methods offer the over-expression of wild-type receptors or the expression of mutant forms that bear a short foreign epitope tag (Table 1), which is usually located in the extracellular space and is recognized by a specific antibody [22-34]. These epitope tags overcome the problem, that suitable antibodies are not available for all GPCRs. Another advantage of anti-epitope antibodies is that antibodies directly recognizing receptor regions may trigger mechanisms, e.g. signal-transduction, internalization or redistribution that are normally activated by the natural ligand or other effector molecules. This might be a disadvantage as the activity of the antibody might influence the cellbiology and read-out of the results. The accuracy of protein recognition depends on the specificity of the primary antibody that should be validated using parallel methods. The tag recognition can be enhanced by introducing more than one repeat of the used epitope [35]. The antigenic epitope can also be useful for other biochemical applications on GPCRs, such as (co-)immunoprecipitation, immobilization and purification.

**Table 1:** Important epitope tags used on GPCRs for IF studies

**Epitope tag**	**Sequence**	**Remark**	**Reference**
HA	YPYDVPDYA	peptide from human influenza hemagglutinin protein	[22-26]
FLAG	DYKDDDDK	synthetic peptide	[27-29]
T7	MASMTGGQQMG	major capsid protein of the T7 phage	[30,31]
c-myc	EQKLISEEDL	human c-myc gene protein	[32]
His_6 _or His_10_	(H)_6/10_	polyhistidine	[33]
VSV-G	YTDIEMNRLGK	vesicular stomatitis virus glycoprotein	[34]

**Figure 2:**
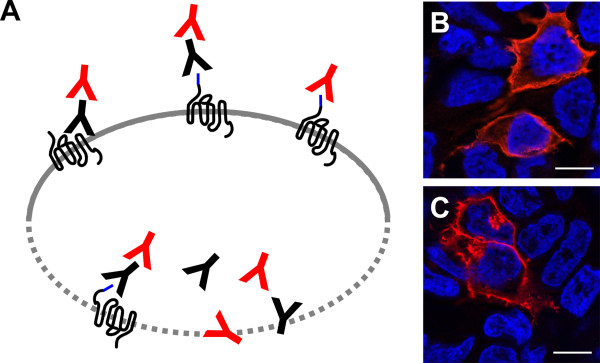
**Visualization of GPCRs by fluorescent antibodies**. Antibodies against extracellular receptor regions or N-terminal epitope tags can be used to visualize GPCRs in non-permeabilized cells (A, above), intracellularly located receptors, intracellular receptor segments or C-terminal epitope tags are only recognized after cell permeabilization (A, below). Either the primary antibody is fluorescent or a secondary antibody carrying a fluorophore is applied in a second step. Localization of the N-terminally HA-tagged human Y_1 _receptor in HEK293 cells (B and C; HA-tagged receptor in red, nuclei in blue, bar represents 10 μm). The receptor can be visualized in the cell membrane of both non-permeabilized (B) and permeabilized cells (C) and intracellularly only within permeabilized cells (C).

In the field of GPCRs immune fluorescence (IF) provides the possibility to visualize the receptors in the membrane of living or fixed cells, either with antibodies against extracellular receptor regions [36-40] or an N-terminally introduced epitope tag. Intracellular receptor segments, C-terminal epitope tags or intracellularly located receptors are only recognized after cell fixation and permeabilization and lead to visualization of membrane-localized and subcellular-localized receptors (Figure 2) [41,42]. For confirmation of results it may also be useful to detect an N-terminal and another C-terminal tag in parallel [43].

#### Auto-fluorescent proteins (AFPs)

The discovery and isolation of the green fluorescent protein (GFP) from the light-emitting organ of the jellyfish *Aequorea victoria *in 1962 and the gene cloning of the complementary DNA (cDNA) initiated the broad use of fluorescence imaging in cell biology [44,45]. GFP is a 238 amino acid protein that consists of 11 anti-parallel β-strands surrounding a central α-helix to form a barrel-such as β-can structure [46]. Its natural function is to convert the blue chemiluminescence of the Ca^2+^-sensitive photo-protein aequorin into green light [47]. The tripeptide chromophore is localized in the centre of the protein and therefore well protected from the environment. The main reason for the success of GFP is its own nature: it is auto-fluorescent and the chromophore is auto-catalytically generated. Thus GFP does not require any additional substrates or co-factors and the fluorescence is not species specific.

The heterologous expression of GFP allows its application as a reporter molecule or the genetic labeling of biomolecules and therefore their direct visualization *in vivo *[48]. Standard genetic-engineering allows the covalent labeling of proteins, subcellular compartments, cells of interest and specific tissue regions by using the protein expression system of the cell. Transfection and transgenic techniques enable the delivery of exogenous DNA more easily than the delivery of dyes to cells and even to whole organisms [49]. However, early variants of GFP were frequently misfolded and led to the aggregation of the fusions. In addition, GFP is a full-sized protein and therefore its fusion may interfere with the expression, function and activity of the protein of interest [50,51]. Creating a successful fusion protein requires the maintenance of the fluorescence of GFP, the functionality of the protein of interest (POI) and the integrity of the chimeric protein. This can be highly dependent on the length and sequence of the linker between GFP and the POI and should be taken into account and optimized for each specific application. To avoid difficulties in protein folding, mostly N- and C-terminal fusions are generated, but the cDNA of the fluorophore can also be integrated into the DNA sequence of many biomolecules [52]. However, GFP turned out to be a rather inert molecule which in most cases did not affect the functional integrity of its fusion partner, which might be explained by its compact molecular structure.

The generation of spectral GFP variants as well as the discovery of novel GFP-like proteins from *Anthozoa *and *Discosoma *(DsRed) [53-61] has significantly expanded the variety of colors available for cell biological applications from the blue to the red range of the visible spectrum (Table 2) and many expression plasmids designed to generate C- or N-terminal fusions with the fluorophore are commercially available [62]. Laboratory mutagenesis has further diversified the spectral properties of fluorescent proteins (FPs), increased their brightness and folding efficiencies and decreased oligomerization [63-66]. These variants allow the simultaneous imaging of different proteins co-expressed as GFP fusions and the fluorescence from at least four analogues can be fully separated through the development of imaging instrumentation with appropriate filter sets or excitation laser lines and software that facilitates linear un-mixing of the fluorescence signals [67,68].

**Table 2:** Most important monomeric GFP variants and their spectral properties

**Protein**	**λ_ex_** **[nm]**	**λ_em_** **[nm]**	**Relative brightness** **[% of EGFP]**	**Relative photostability** **[t_1/2 _in s] **[66]	**Reference/source**
**Blue fluorescent proteins**					
T-Sapphire	399	511	79	25	[53]

**Cyan fluorescent proteins**					
mCFP	433	475	39	64	[54]
Cerulean	433	475	79	36	[55]
CyPet	435	477	53	59	[56]

**Green fluorescent proteins**					
EGFP	484	507	100	174	BD Biosciences Clontech
Emerald	487	509	116	0.69	[48], Invitrogen

**Yellow fluorescent proteins**					
EYFP	514	527	151	60	[48], Invitrogen
Venus	515	528	156	15	[57]
mCitrine	516	529	174	49	[58]
YPet	517	530	238	49	[56]

**Orange and red fluorescent proteins**					
mKO	548	559	92	122	[59], MBL Intl.
mOrange	548	562	146	9	[60]
tdTomato	554	581	283	98	[60]
DsRed-monomer	556	586	10	16	BD Biosciences Clontech
mStrawberry	574	596	78	15	[60]
mCherry	587	610	47	96	[60]
mPlum	590	649	12	53	[61]

**Figure 3:**
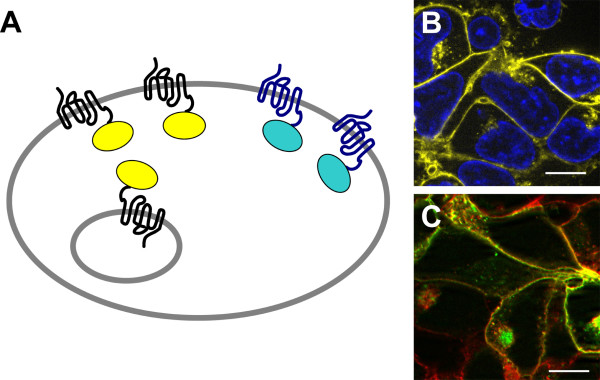
**Visualization of GPCRs by auto-fluorescent proteins**. Commonly, AFPs are fused to the C-terminus of the GPCR (A). Localization of hY_1_R-EYFP in HEK293 cells (B; receptor in yellow, nuclei in blue, bar represents 10 μm). Receptor expression as well as membrane and subcellular GPCR localization can be easily followed, since the fluorescence is generated *in vivo *and no additional labeling is reqired. Localization of human Y_5 _receptor C-terminally fused to ECFP in HEK293 cells stably expressing hY_1_R-EYFP (C; hY_5_R in green, hY_1_R in red, bar represents 10 μm). The spectral properties of AFPs allow receptor co-expression and co-localization studies.

Fluorescent proteins are a powerful tool for the investigations of GPCRs in living cells (Figure 3). Many GPCR systems have been studied so far by using GFP or its variants, since this is generally the first method of choice for non-invasive imaging in order to monitor gene expression, subcellular distribution and trafficking [62].

The development of advanced auto-fluorescent proteins, such as photo-activatable, photo-switchable and photo-convertible AFPs [69], fluorescent sensors [70], timer [71,72] and split AFPs [73] provides novel applications for fluorescent labeling *in vivo *for studying the biosynthesis, expression, localization, movement, activity and turn-over of proteins as well as the direct measurement of cellular parameters and organelle functions at the single cell and even down to the single molecule level [74].

With the development of resonance energy transfer (RET) techniques such as FRET [75,76], bioluminescence resonance energy transfer (BRET) [77] and the bimolecular fluorescence complementation (BiFC) [78,79] approach the opportunity arose to gain insights into protein-protein-interactions in living cells by using the appropriate pairs of autofluorescence proteins.

#### Peptide- and protein-tags

The search for alternatives to AFPs led to the ongoing development of chemical labeling strategies for the selective and site-specific coupling of fluorophores to genetically encoded peptide- or protein-tags [80-90], which expanded the utility of *in vivo *protein imaging (Table 3) [91].

**Table 3:** Important peptide- and protein-tags for fluorescently chemical labeling applied on living cells

**Tag**	**Remark**	**Labeling agents**	**Reference**
**Lumio™-tag**	tetracysteine/biarsenical system tetracysteine motif: CCXXCC size: 6–20 aa	biarsenical dyes, e.g.: CHoXAsH (blue) FlAsH (green) ReAsH (red) membrane-permeable non-fluorescent until binding	[80,81,93]

**His-tag**	oligo-histidine/nickel-complex system oligo-histidine sequence: (H)_n_, n = 6 size: 6–10 aa	nickel-nitrilotriacetic acid (NTA-Ni^2+^) dyes, e.g.: NTA-FITC-Ni^2+ ^(green) NTA-QSY-Ni^2+ ^(red) NTA-DCF-Ni^2+ ^(green)	[82,83]

**SNAP™-tag**	mutated human O^6^-alkylguanine alkyltransferase (hAGT) size: 182 aa	fluorescent benzylguanine (BG) derivatives, e.g.: BG-505 (green) BG-TMR-*Star *(red) cell-permeable or cell-impermeable	[80,84,85]

**ACP/PCP-tag**	acyl carrier/peptide carrier protein tag labeled via phosphopatheine transferase (PPTase) size: 80 aa	fluorescent coenzyme A conjugates, e.g.: CoA-fluorescein (blue) CoA-Cy3 (green) CoA-Cy5 (red) cell-impermeable	[86,87]

**HaloTag™**	mutated prokaryotic dehalogenase size: 297 aa	fluorescent haloalkane ligand, e.g.: HaloTag 488 ligand (green) HaloTag TMR ligand (red) cell-permeable or cell-impermeable	[88,89]

**AP-tag**	biotin acceptor peptide labeled via *E. coli *biotin ligase BirA size: 15 aa	fluorescent streptavidin, e.g.: streptavidin-Alexa568 (red)	[90]

Concerning the binding mechanism most labeling techniques can be classified into two major categories: affinity labeling and enzymatic labeling. Affinity labeling is based on a non-covalent chelation and provides a simple and highly selective labeling procedure that is applicable to various sites within the POI. On the other hand, irreversible covalent labelling by enzyme-catalyzed labeling methods still is more suited for the clear analysis of the POI, since these methods provide a higher stability. Therefore non-enzymatic covalent labeling is highly attractive since there is no need for a large enzyme or a protein domain and many labeling reagents as well as reaction conditions are well suited to this strategy.

In most cases self-labeling tags are smaller in size than AFPs and can be post-translationally labeled with a variety of synthetic fluorescent probes. These can provide alternative spectroscopic properties compared to AFPs [92]. They can be advantageous in applications, if the size of the tag is important [93] or if the conditions are not suited for AFPs, such as anaerobic environments [94]. Self-labeling tags are also a promising addition to the tools available for the immobilization and purification of proteins via affinity chromatography and can be useful in microarrays or on beads for pulldown assays.

**Figure 4:**
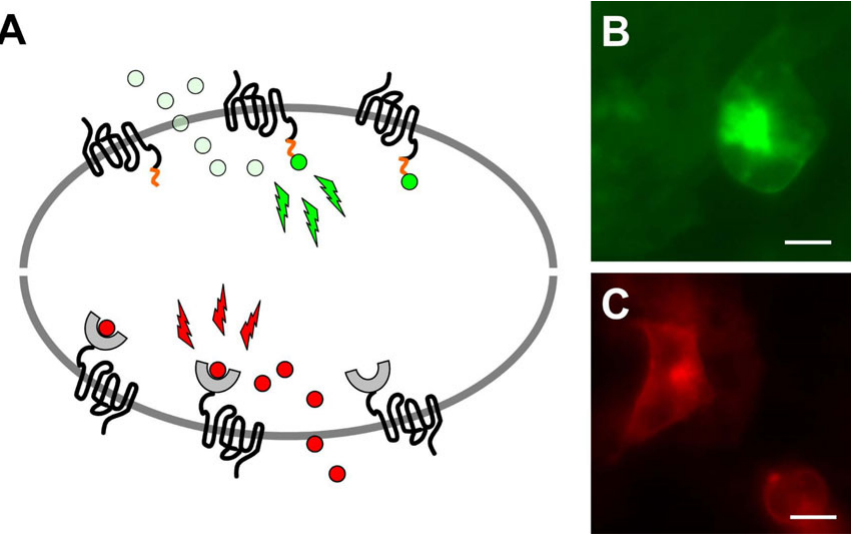
**Visualization of GPCRs by peptide- and protein-tag technologies (A; above: Lumio™-tag technology and below: SNAP™-tag technology)**. Membrane and subcellular localization of human Y_1 _receptor C-terminally fused to the tetracysteine motif in HEK293 cells and labeled with the membrane-permeable FlAsH dye, which fluoresces green after binding to the motif (B; bar represents 10 μm). Cellular distribution of hY_1_R C-terminally modified by the mutant hAGT in HEK293 cells and labeled with the membrane-permeable, red fluorescent BG-TMR-*Star *dye (C; bar represents 10 μm).

Chemical labeling of fusion proteins has the advantage that the selectivity of labeling is genetically encoded, but the fluorescent properties of the probes can be modified synthetically (Figure 4). Since most fluorescent probes are membrane-permeable the POI can be labeled nearly at every site of the molecule. Labeling systems with cell membrane impermeable probes are only suited for cell-surface labeling applications.

The opportunity to control the localization and the time point of labeling enables scientists to study protein function in time and space inside a living cell at the molecular level. The choice of using different dyes at various time points during the experiment in cells, when protein translation is not inhibited, will lead to distinct populations of otherwise identical proteins whose discriminating features are determined through the time point of the respective labeling of each population. Such pulse-chase-experiments will reveal further insights into protein function and localization.

#### Fluorescent ligands

The history of fluorescent ligands has been followed by the development of commercially available fluorophores (Table 4) [16]. Low molecular weight organic dyes have been designed and synthesized to exhibit excitation and emission wavelengths that are tuned to the excitation sources of the fluorescence signal readout instrument. These dyes can be coupled by their functionalities in easy to handle conjugation reactions. The most widely used labels are based on xanthene dyes or the cyanine structure [95-104]. Succinimidyl esters have become the preferred reactive group for labeling of amino groups and lead to the formation of stable peptide bonds. This reaction is easy to control, in contrast to the reaction of sulfonyl chlorides. Maleimide and iodoacetamide derivatives represent the state-of-the-art for the labeling of sulhydryl groups.

**Table 4:** Fluorescent dyes to label receptor ligands

**Ligand**	**Action**	**Receptor**	**Reference**
**Fluorescein dyes:**	cover green wavelengths of the visible spectrum contra: photo-bleaching, pH sensitivity		
Fluorescein-naloxone	antagonist	MOR	[95]
Carboxyfluorescein-NPY	agonist	YR	[96]

**Rhodamine dyes:**	cover red wavelenghts of the visible spectrum pro: pH insensitivity, photo-stability contra: water solubility, non-specific binding, quenching		
Rhodamine-angiotensin II	agonist	ATR	[97]
TRITC-α-bungarotoxin	antagonist	AChR	[98]

**Cyanine dyes:**	cover full spectrum from UV to IR pro: water solubility, reduced quenching, diverse reactive groups		
Cy3-EGF	agonist	EGFR	[99]
GR119566-Cy5	antagonist	5HT_3_R	[100]

**BODIPY dyes:**	cover visible range pro: sharp excitation and emission, high and insensitive quantum yields, pH insensitivity		
BODIPY FL-hPP	agonist	Y_4_R	[101]
BODIPY 558/568 pirenzepine	antagonist	M_1_R	[102]

**Alexa fluor dyes:**	cover spectrum from visible range to IR pro: pH insensitivity, photo-stability, increased brightness, diverse reactive groups		
Alexa 532 adrenaline	agonist	β_2_-AR	[103]
Alexa 647 CXCL11	agonist	CXCR	[104]

**Quantum dots:**	pro: broad absorption, narrow and tune-able emission, photo-stability, strong luminescence, long luminescent lifetimes, multivalent surface contra: large size		
Deltorphin II(Ile-Ile)-QD595	agonist	DOR	[107]
QD560-dopamine	agonist	D2R	[108]

There is a remarkable trend to develop and use labeling reagents that fluoresce at longer wavelengths, which allow the measurement of still more parameters within multi-color experiments and are suited for fluorescence imaging *in vivo *[105]. The most significant advantage of these dyes is the reduced background fluorescence from cells, cell debris, buffer components and plastic materials. However, infrared fluorescent labels have a lower chemical and photo-stability. Semiconductor nanocrystals, called quantum dots (QDs), provide a great alternative to traditional dyes [106]. They fluoresce throughout the visible and near-infrared spectrum and can be excited very efficiently with one excitation source. Since they possess narrow emission bands, up to 20 QD reagents could be detected separately with narrow band-pass filters. Therefore they will also promote the applications of fluorescently labeled peptide ligands in the future [107,108].

Besides its pharmacology, the fluorescent ligand has chemical properties that will determine its behavior on and in a cell. Most drugs can be placed within a spectrum of lipophilicity to hydrophilicity. It is important to determine these properties and if they are altered by the addition of a distinct fluorescent molecule dependent on its position [109]. Therefore preference should be given to fluorophores with high stability and fluorescent yield as well as resistance to photo-bleaching and which preserve the pharmacological properties of the ligand.

Many fluorescent ligands are peptides. The synthesis of fluorescent small-molecule ligands of GPCRs is not a trivial process. A potential site for fluorophore conjugation is in much closer proximity to the pharmacophore for small molecules and as a consequence, much more likely to affect ligand affinity and efficacy. A common approach includes the separation of the ligand and the fluorophore by some form of linker or spacer, which may vary in length and chemical nature as requested by the biological activity. Well established high-affinity ligands with excisting structure-activity relationship data can suggest whether a modification of a chemical site in the molecule might be tolerated. Positional scanning peptide combinatorial libraries can also be used to identify new fluorescent ligands [110] and procedures such as the Macro-model's large-scale low mode (LLMOD) enable the conformational profiling of fluorophore-modified peptides [111].

**Figure 5:**
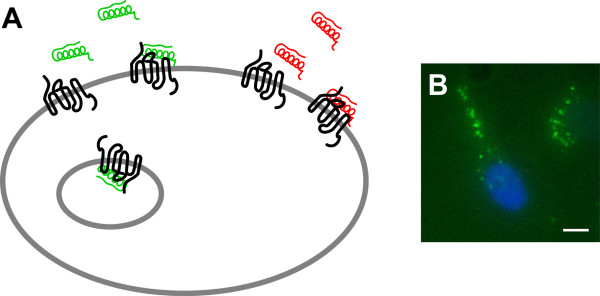
**Visualization of GPCRs by a fluorescent agonist (A; green) or a fluorescent antagonist (A; red)**. Fluorescent agonists induce receptor internalization to intracellular endosomes as demonstrated by SK-N-MC cells, endogenously expressing the human Y_1 _receptor, incubated with CF-labeled neuropeptide Y (B; NPY in green, nuclei in blue, bar represents 10 μm). In contrast, fluorescent antagonists prevent receptor trafficking to intracellular compartments and target receptors at the cell membrane as shown by Schneider et al. using MCF-7 cells, endogenously expressing the Y_1 _receptor, incubated with Py-1-labeled Y_1_R antagonist derived from BIBP3226 [112].

Fluorescent ligands have so far been developed for a variety of GPCRs to investigate ligand-receptor interactions. The ligand to be tagged may be an agonist or an antagonist (Figure 5). Antagonists usually offer a higher affinity and thus provide a better signal to noise ratio than agonists [112], but in most cases they do not induce receptor internalization, although receptor clustering was observed [113]. Metabolically stable analogs may prove more advantageous *in vivo*, however their intracellular fate may not faithfully mimic that of the native ligand.

### Fluorescence applications to study GPCRs

The described methodologies to label GPCRs provide the possibility to monitor the expression and cellular localization of these biomolecules. But visualization represents only the first step of a variety of colorful applications for unraveling biological processes. The investigation of GPCR functionality, clustering, trafficking, biosynthesis and degradation as well as the identification and visualization of important protein-protein-interactions, such as receptor oligomerization, ligand binding, G-protein coupling and arrestin recruitment in living cells has become feasible. Recent advances in fluorescence instrumentation not only allow qualitative but also quantitative data analysis and led to the ongoing development of high-throughput applications.

#### Expression and localization

Fluorescent antibodies and ligands as well as auto-fluorescent proteins and self-labeling tags are suited for monitoring receptor expression and cellular distribution. However, some considerations due to the difference in the labeling procedures have to be taken into account to receive reliable results.

Immunohistochemistry is a valuable method to localize GPCR expression and provides important information for defining receptor function and disease association. Fluorescent antibodies can be used to visualize the tissue and cellular distribution of receptors with a far greater lateral and axial resolution than offered by autoradiography [114,115]. The fluorescence can be quantified by fluorescence activated cell sorting (FACS) analysis or by special microscopic software. A limitation of the immunofluorescence method is the low expression level of wild-type receptors in normal cells and native tissues. One early method, which was developed to overcome this problem, is the tyramide signal amplification (TSA) method [116]. This is a sensitive immunodetection technique based on the peroxidase (POD) catalyzed deposition of labeled tyramide molecules. TSA in both direct (fluorophore conjugated) and indirect (biotin or dinitrophenol conjugated) variants has been used to amplify signals. It was furthermore successfully applied in double and triple labeling immunofluorescence confocal-based studies [117] and allows the use of low antibody concentrations to reduce background and non-specific binding.

Since receptors are highly mobile within the cell and the membrane, IHC generally requires fixation of cells and tissues. Therefore, this approach can be used to detect receptor localization at fixed time points however chemical fixation may negatively translocate them on the cellular level. It is very important to choose a fixative with relatively mild shrinkage effects on cells, as well as non-fluorescent properties to avoid cross-talk of signals between the chemical and the applied fluorophore(s). The fixation of cells could also negatively affect the conformation of the receptor antigen and thus the antibody binding. The conditions of fixation (temperature, time, pH) are furthermore critical for the accessibility of the antigenic epitope [118]. Since the antibodies are not able to pass the cell membrane, IF is usually restricted to the extracellular receptor site. Only cell permeabilization enables the receptor detection at the intracellular receptor site or in subcellular compartments.

However, labeled antibodies provide the possibility to quantify the surface expression of receptors, because the antigenic epitope can also be detected via a cell surface ELISA [119]. In this approach the secondary antibody is labeled with an enzyme, e.g. peroxidase (POD) or alkaline phosphatase (AP) that catalyzes a reaction, which results in a chromogene product that is easily measurable with a plate reader. This application is widely used to compare the surface expression of different receptor subtype mutants [120]. Using permeabilized cells in a parallel approach the ratio between total and surface expressed receptors can be determined. Additionally, both populations can be visualized e.g. with species-specific primary and different fluorescent secondary antibodies [121].

Fluorophore-tagged GPCR ligands allow the direct non-radioactive visualization of their receptor target at the single-cell level, provided that the affinity and selectivity of the modified ligand is known not only at recombinant cells over-expressing a particular receptor but can also lead to the endogenous receptor. With respect to improved stability, detection of different receptor subtypes in cells and tissues and prevention of receptor internalization small non-peptidic fluorescent antagonists should be applied. It should be noted, that the choice of the fluorophore cannot only variably influence the host pharmacophore but also can lead to different fluorescence distribution patterns in receptor expressing cells [122]. Since it is impossible to predict exactly how a fluorescent drug analogue will perform or distribute within a living cell careful pharmacological, chemical and biological validation of the fluo-ligand are required. Compounds that only show fluorescence when they are bound to the receptor offer low background fluorescence in the aqueous phase and thus a high signal to noise ratio. Novel methodologies for studying receptors in native cell tissue or recombinant receptors in cell culture were developed and validated [123] and used for the identification and localization of receptors in primary cultures of native cells [9,124] and also in intact blood vessels [125,126].

Sufficient washing steps or quenching procedures are required to receive clear images of GPCR expression and localization and to specifically detect the GPCR signal. This can lead to complications, such as influencing cell viability or to promote ligand dissociation. The commonly first and simplest method to monitor GPCR expression and localization is the genetic generation of GFP receptor fusions. Because GFP is covalently attached to the protein of interest potential problems with non-specific fluorescence can be avoided. GPCR-GFP fusions can also overcome problems of relatively low levels of receptor expression, the stoichiometry of the receptor and the fluorescent protein is well defined. These fusions offer enhanced sensitivity and resolution in comparison to standard antibody staining techniques and there is no distortion or alteration of membrane compartments, since there is no need for cell fixation, cell permeabilization or additional labeling steps [127]. However, cells can be fixed for convenience, because GFP is chemically resistant. Additionally, GPCR-GFP fusions are more resistant to photo-bleaching than antibodies or ligands. They exhibit low background fluorescence and permit kinetic studies of protein localization and trafficking. This allows investigators to visualize proteins for a longer duration of time in an intact cellular environment than currently possible with the use of other extrinsic fluorescent probes. The real time expression in living cells can be easily detected and quantified by fluorescence microscopy, fluorescence spectroscopy, FACS analysis or fluorometric assays. Recent advances in instrumentation and image analysis have opened the door to high-throughput *in vivo *studies that can provide the morphological and temporal context for the biochemical pathways regulating cell function [128].

As mentioned above it is very important to check, whether the fluorophore-tagged receptor retains its natural properties, because it can be important to either label the receptor at its N- or C-terminus. Labeling can negatively influence the ligand binding pocket or the coupling of the G-protein in the signal transduction process. Thus, careful experimental comparisons to untagged receptors should be made whenever possible. For successful fluorescence detection enhanced and optimized monomeric GFP variants should be applied, since the formation of GFP oligomers can result in aggregation of the chimera and disturbance of the target protein function and localization.

Being fused to a GPCR the GFP fluorescence is expected to be well located within the cell membrane but possibly also to certain amounts in subcellular compartments, such as the endoplasmic reticulum (ER) or the Golgi apparatus (GA), because the chimeric protein in living cells reflects the normal turn-over of the GPCR. Thus, the surface expression of a given GPCR can be easily monitored but this might also depend on the cell system used, because the protein repertoire of the cell line might have an influence on the expression, localization and distribution of the GPCR-GFP chimera [129]. Therefore, it is important to always combine visualization with functional studies, in order to identify the possible role of intracellularly located receptors and their relationship to surface membrane-located proteins. Appropriately membrane-located GPCRs can also be used to screen for mutants to identify regions within the receptor that are important for subcellular targeting [130].

Despite the rapid progress in visualization of GPCRs in living cells caused by auto-fluorescent proteins these illuminators have limitations. They are relatively bulky, therefore interference with protein structure, localization and function may occur. Genetically encoded peptide- and protein-tags for chemical labeling provide an alternative method to label and visualize receptors in living cells, thereby reducing the size of the receptor-fluorophore complex and expanding the spectral range to the near-infrared region.

However, there is great variation among these methods in terms of labeling specificity, velocity, stability, size of the tag, toxicity and versatility to probe structure and cell type and no single method yet succeeds in all these respects. Unspecific labeling within the cells has to be avoided for a specific detection. Therefore washing steps are indispensable to reduce possible background fluorescence. Systems providing dyes that are non-fluorescent before binding, e.g. the Lumio™-tag technology, or that perform a spectral change while binding to the tag positively influence the signal to noise ratio. Another important consideration for chemical labeling in living cells is the potential of the probe or targeting sequence to affect the cellular system via toxicity or more complicated interactions, which have to be ruled out by suitable controls and improved labeling protocols. The stability of the fluorescent complex is influenced by the possible dissociation of the fluorescent probe as well as the degradation of the complex. The labeling timescale will define the number of biological processes that can be studied, the faster the labeling, the more processes may be addressed. However, these self-labeling tags can be used to differentiate easily between intracellular and extracellular pools of the membrane localized proteins, e.g. GPCRs. For this application the tag has to be introduced at the N-terminal of the receptor site and a tag system has to be applied that provides both cell-permeable as well as cell-impermeable labeling dyes.

#### Cell surface dynamics and mobility

Increasing evidence favors the concept of membranes being organized into domains with defined lipid and protein compositions. These domains are believed to serve as platforms for trafficking, sorting, signalling and pathogen entry by concentrating certain selected lipids (e.g. cholesterol and sphingolipids) and proteins [131-133]. Resistance to solubilization by mild non-ionic detergents at low temperature represents an extensively used biochemical criterion to identify, isolate and characterize those membrane domains [134]. Detection of proteins in detergent-resistant membranes is usually performed either by immunoblotting or ligand binding. However, these methods are not suitable in cases in which ligand binding is compromised in the presence of the detergent and/or is limited by the availability of antibodies with high specificity. GFP tagged membrane proteins are an alternative to directly determine detergent insolubility of GPCRs based on the fluorescence quantification of the membrane protein before and after detergent treatment [135]. Specific lipid (DiIC_16 _and *FAST *DiI) and protein (transferrin receptor) markers have been utilized to validate this fluorescence approach. The method of analysis of detergent insolubility can be useful in exploring localization and organization of GPCRs in membranes and has the potential to be used in large-scale screening as well.

The activation of GPCRs leads to the recruitment and activation of heterotrimeric G-proteins and occurs at the plasma-membrane. The lateral mobility of the activated receptor on the cell surface represents an important prerequisite for the interaction with G-proteins and has a significant impact on the overall efficiency of the signal transduction process [136,137]. To examine the cell surface dynamics of a GPCR in the plasma membrane the fluorescence recovery after photo-bleaching (FRAP) technique can be applied [138]. This method involves the generation of a concentration gradient of fluorescent molecules by irreversibly photo-bleaching a fraction of fluorophores with a high intensity laser in a small area of the cell membrane. The recovery of fluorescence into the bleached region is used to measure the membrane diffusion characteristics of the GPCR, but also their changes in terms of ligand binding, activation as well as receptor oligomerization [139-141].

The application of confocal microscopy and multiple fluorophore-tagged ligands can provide the basis for time course studies of receptor cluster formation [100]. The receptor mobility can be visualized by photo-dissociation of a fluorescent ligand – a process distinct from photo-bleaching. The ability of intense focused light to remove a fluorescent ligand from one site and hence allow the binding of a ligand molecule of another color enables the visualization of the movements of spatially restricted subpopulations of cell surface receptors [142].

Comparing FRAP and FCS, both methods can be used for different applications. Whereas FCS can only be used to monitor mobile receptors, FRAP also allows to follow immobile molecules. In contrast, only FCS can be used to provide information on a single molecule level.

#### Trafficking – internalization and recycling

In response to agonists GPCRs desensitize, aggregate on the cell surface and move from the plasma membrane into intracellular vesicles at different rates and to varying extents. Then, GPCRs are either recycled to the cell surface or degraded in lysosomes [1,2].

Classically, receptor internalization after ligand exposure has been measured by radio-ligand binding and laborious physical fractionation techniques. This process can also be visualized and quantified by immunofluorescence studies. One approach is the measurement of the loss of surface receptors from the cell membrane as a result of internalization. In this case the receptors are not accessible to antibodies from outside the cell anymore. Receptor sequestration is then defined as the fraction of total cell surface receptors that, after ligand treatment, are removed from the cell surface [143]. Otherwise it is also possible to detect the internalized receptors inside the cells in comparison to un-stimulated examples. Accordingly, the receptors have to be labeled with the antibodies prior or after ligand exposure. Since cell fixation and permeabilization are a prerequisite for receptor detection after ligand treatment, the labeling with antibodies within this application can not be performed in living cells. The receptor labeling before the stimulation provides the detection of receptors available on the cell surface at a certain time point but requires the ability of the ligand to bind to the bulky antibody-tagged receptor compared to the un-tagged one. The bound antibodies should not interfere with the ligand binding process and for that reason fluorescently labeled primary antibodies may facilitate this approach by a smaller antibody-receptor-complex. If there is no fluorophore-labeled primary antibody available, it will be a compromise to perform the receptor stimulation after labeling with the un-labeled antibody and thus visualize it with the labeled receptor after fixation and permeabilization.

Labeling of cell surface receptors with fluorescent antibodies at a specific time and the subsequent ligand exposure provides the basis for receptor recycling experiments in living cells by applying either fluorescence microscopy, FACS or ELISA. If the labeled GPCR recycles back to the cell surface after the removal of the ligand then these defined GPCRs will be detected and quantified in the cell membrane again, in contrast to only stimulated or degraded receptors [43]. Receptor recycling can then be defined as the fraction of total cell surface receptors, after ligand treatment and clearance of stimulus, that are back at the cell surface and therefore accessible for antibodies [144-146]. But without receptor pre-labeling, also un-stimulated and membrane-destined, recruited and recycled receptors can be co-detected with the stimulated and recycled receptors as a separate second population.

N-terminally located self-labeling tags and their corresponding membrane-impermeable dyes can also serve as a tool for studying GPCR internalization and recycling, since only the labeled receptors are visualized prior to the ligand stimulation. In contrast to antibodies, the size of the GPCR-fluorophore complex is significantly reduced. However this approach also requires ligand binding to the pre-labeled receptor that is not interfered with by the label.

The use of GFP-tagged receptors enables a more simple and rapid assessment of GPCR trafficking, which is not possible by other techniques. The ligand can be applied to living cells and the movement of GPCRs can be visualized in real-time under varying conditions concerning incubation time, temperature as well as ligand concentration and specificity. The internalized GFP-labeled receptors are visualized as numerous punctuated spots in the perinuclear region of the cell. Algorithms have been developed that identify and collect information about these spots, allowing the quantification of the internalization process and the screening of ligand-induced receptor dynamics in whole cells. The obvious advantage to pharmacologists, in using GFP fusion proteins over antibodies is the ability to promote receptor sequestration by using both, agonists and inverse agonists [147]. With the help of fluorescent markers for subcellular compartments, e.g. for endosomes (transferrin) or for lysosomes (dextran, LysoTracker), the GPCR can be directly located within the cell and its recycling back to the cell membrane and/or degradation can be easily detected. GFPs that are more sensitive to lower pH levels, such as the "ecliptic pHluorin" or the enhanced yellow fluorescent protein (EYFP), can be used a non-invasive pH indicator for intracellular organelles and cytoplasm, and for the quantification of GPCR trafficking, because the fluorescence is quenched as these protein chimeras enter lysosomes [148,149].

Since only agonist ligands seem to promote receptor internalization, also fluorescent agonistic ligands are a valuable tool for the investigation of receptor trafficking, not only in living cell culture studies but also in living neuronal cells. The accumulation of fluorescent ligands at the perinuclear region appears to be a common feature of many GPCR-ligand complexes [150]. To study the cellular receptor distribution after agonist exposure it is necessary to remove unspecifically bound molecules as completely as possible. Frequently applied methods include hypertonic acid stripping that removes surface-bound ligand while leaving the intracellularly bound sites for analysis [151]. The fluorescent yield of some dyes was found to be pH sensitive, an effect that possibly is potentially helpful in internalization studies by indicating the presence of GPCRs in endosomal compartments with relatively high pH. The fate of both, receptor and ligand can be simultaneously determined and visualized when a fluorescent ligand and a fluorophore labeled receptor are used together [97,129]. Studies have examined the trafficking of the ligand as well as of the receptor, which provides further important insights into the fate of the receptor-ligand complex as well as into cellular mechanisms such as the regulation of the GPCR signal via ligand degradation [129].

Of course the value of the fluorescent methods has critically to be compared with classical biochemical methods, e. g. co-immunoprecipitation and fractionation. A clear advantage of the fluorescent methods is their application on whole cells as well as the possibility to monitor the effects in a time-dependant manner. Limits, however, are due to cross-talk, channel bleed-through and limited resolution because membrane microdomains usually are below light resolution.

#### Biosynthesis

GPCR biosynthesis, folding and assembly take place in the endoplasmic reticulum (ER). When the receptors are correctly folded, they are packaged into ER-derived vesicles and migrate from the ER to the ER-Golgi intermediate complex (ERGIC), the Golgi apparatus and the *trans*-Golgi-network (TGN). During this transport process receptors undergo post-translational modifications (e.g. glycosylation) to ensure final migration to the cell surface [152]. The export from the ER and the membrane targeting are highly regulated processes and the detailed mechanisms are not explicitly understood. These investigations will need further information on the age, expression, lifetime and movement of GPCRs also for the comparison of ligand-stimulated and un-stimulated GPCR-expressing cells. Therefore, it is indispensible to apply methodologies which can distinguish between old and newly synthesized receptors in living cells.

In order to discriminate between different populations of membrane located GPCRs pulse-chase studies can be performed with different fluorescent variants of the used antibody. This application on living cells can provide new insights into the receptor turnover, but is dependent on the applicability of primary fluorescent antibodies. In terms of investigating receptor biosynthesis after ligand stimulation again the appropriate ligand binding to the antibody-receptor complex is necessary. Intracellular receptors have not yet been delivered to the plasma membrane and receptors present at the plasma membrane have their first N-terminal epitope irreversibly cleaved by the enzyme [153].

Receptor-specific fluorescent ligands with high binding affinity and low off-rates have also been found to be suited for receptor pulse labeling [100]. The low off-rate of the ligands ensures the stability of the ligand-receptor complex during the whole period of the experiment. Different chromophores attached at the same pharmacophore enable the consecutive application of spectroscopically distinguishable ligands to visualize the surface appearance and turnover of GPCRs.

The development of fluorescent protein variants opens up the possibility of performing "pulse-chase" experiments in living cells, by visualizing a distinct pool of the protein of interest in a defined region of the cell and following its transport and turn-over in real time. Important advances include the development of GFP-variants that can be activated by ultra violet (UV-) light, such as photo-activatable GFP (PA-GFP) [154], or those that can change their color from cyan to green [155], or from green to red [156] upon intense illumination with violet or UV-light. However, the complex photochemical processes underlying the phenomena of photo-activation and photo-conversion can also cause problems with normal fluorescent proteins leading to potential artifacts under certain circumstances. Intense illumination of EYFP with 514 nm laser light can lead to photo-conversion to a protein with ECFP-like fluorescence properties, which can be a problem in experiments that are based on bleaching of EYFP [157]. Photo-bleaching that leads to photo-toxicity can be significantly reduced by specialized forms of confocal microscopy such as spinning-disk microscopy, in which the excitation light is guided through a series of small pinholes [158] or by excitation technologies that are applied in two-photon or multi-photon laser scanning microscopy [159].

However, the recently established self-labeling tags are and will be suited for pulse-chase studies more efficiently than all other fluorescence technologies. The free choice of specific time points for pulse labeling of the receptor and the sequential labeling of receptor subpopulations with spectrally distinguishable fluorophores provide a promising tool for the imaging of consecutively expressed GPCRs and their spatiotemporal organization [160].

#### Functionality – ligand binding and signal transduction

Since GPCRs are associated with diseases they are a very important target for the pharmaceutical industry. The ability to measure and quantify the binding of ligands to these receptors and the obtained responses has been, and remains, a key element of the drug discovery process. The most common way to study this include radioactively labeled drug molecules to label receptors directly on the cell surface or in membrane fragments from cells over-expressing the receptor of interest.

Because of high-throughput and high-content drug discovery assays with improved detection efficiencies, increased health, safety and disposal issues associated with the application of radio-ligands, there is a need to develop more robust fluorescence-based techniques and receptor-specific ligands with fluorescent properties. Fluorescent ligands have several advantages over traditional radio-ligand binding techniques [161] and studies with different neuropeptides comparing radio-ligand with fluorescent ligand binding revealed a higher resolution with fluo-peptides [96]. The interest and use of fluorescent ligands is growing not only to reveal novel information on the life cycle of the receptors but also to develop receptor binding peptides, e.g. small molecular weight antagonists, for diagnosis as well as for therapy [162].

The application of flow cytometry can be used to characterize the specificity of fluorescent ligands [104]. Within this method the applied laser beam is precisely directed towards the surfaces of the receptor-expressing cells, which are centered in the core of a thin sample stream. Therefore it is mainly cell-associated fluorescence that is detected and the signal is hardly impaired by free fluorescent ligand in solution thus allowing measurements of ligand binding under equilibrium conditions [163]. Several innovative approaches in flow cytometry to investigate GPCRs have been described. The determination of binding and/or functional data with intact cells as well as the potential of flow cytometric techniques in high-throughput screening will further advance and accelerate the drug discovery process through experimental setups to gain equilibrium binding, selectivity data and the functional activity of GPCR ligands in one single step [164].

Cell-based FCS measurements with fluorescent ligands can be applied to determine the properties of ligand-receptor complexes within small areas of the cell membrane by measuring the fluctuations of fluorescence intensities and employing mathematical correlation algorithms [165]. Since FCS can determine the diffusion rate of a tagged receptor directly and yield quantitative information about its membrane environment it can provide important insights into subcellular quantitative GPCR pharmacology [166].

Instead of the direct measurement of the fluorescence of the bound or free fluorescent ligand after separation, fluorescence polarization has been identified as a useful method to follow receptor bound ligands even in high throughput assays. As some of the organic fluorescent molecules are sensitive to their surrounding the measurement of the anisotropy or polarization may depend on whether the ligand is bound to the receptor or free. This method has been used to investigate ligand binding of different GPCRs, including vasopressin, melanocortin, neurotensin and opioid receptors [167].

Ligands carrying two fluorophores with spectral characteristics, that are well-suited for FRET measurements, can provide further insights into the bioactive conformation versus the conformation in solution of the ligand by changes in intramolecular FRET due to distance change between the fluorescently labeled residues [168].

Because GPCRs constitute excellent putative therapeutic targets, identification of their endogenous ligands has a great potential for drug discovery. The expression of GFP-tagged GPCRs followed by incubation of the transfected cells with fractions purified from tissue extracts and imaging of ligand-induced receptor internalization has become very important in functional characterization of orphan receptors. The GFP-based internalization assay provides a highly specific quantitative cytosensor technique with sensitivity in the nanomolar range to identify natural and synthetic ligands for GPCRs. Additionally, further improvements in GPCR antibody technology led to conformation state-sensitive antibodies that can also be useful for the identification of molecules with therapeutic interest [169].

The activation of GPCRs is traditionally measured either by membrane-based biochemical assays or by monitoring downstream physiological events. Fluorescence spectrophotometers are widely used for the quantification of photometric and fluorometric cell-based assays, e.g. for the investigation of second messenger systems as an indirect way of assessing receptor function.

Since these methods are not suited for detailed kinetic or spatial analysis of receptor activation and signaling, several optical techniques have been developed to monitor receptor activation continuously and in real-time. These provide new insights in both the mechanistic basis of the signaling process and the kinetic and spatial properties of GPCR-mediated signals [170]. Fluorescent or luminescent labeled ligands, receptors and G-protein subunits, in combination with the development of FRET and BRET approaches, has allowed the determination of kinetic parameters for many steps of the signaling process, including ligand binding [171,172], receptor activation [173,174], receptor-G-protein interaction [175,176], G-protein activation [177] and effector activation [178]. Moreover, sensors have also been developed and further optimized to measure second messenger molecules such as phosphatidylinositol-3,4,5-trisphosphate (PIP_3_) [179], cyclic adenosine monophosphate (cAMP) [180,181] or cyclic guanosine monophosphate (cGMP) [182,183]. FRET sensors are usually fusion proteins of ECFP and EYFP or EGFP and a monomeric red fluorescent protein linked by a sensory domain. This domain is responsive to changes in distinct cellular parameters by a conformational change, leading to a change of the FRET signal. A variety of enzymatic or biological activities can be determined by the appropriate choice of the sensory domain and are also relevant for studying intracellular processes, as well as processes at the cell surface. Despite the development of FRET based sensors, which measure changes between two GFP variants to assess second messengers, sensors have also been developed which measure changes in fluorescence intensity of a single circularly permuted fluorescent protein (cpFP), e.g. for Ca^2+ ^sensing [184].

When GPCRs bind agonists, they are thought to change into an active conformation, which in turn binds to and activates G-proteins. To study this receptor activation by FRET fluorescent probes can be inserted in the third intracellular loop and the C-terminus, respectively. In addition to the most common ECFP/EYFP receptor sensors an ECFP/FlAsH sensor was recently developed [93]. Although labeling with FlAsH requires an extra step and the resultant receptor sensor bleaches more rapidly, similar results have been obtained and the ECFP/FlAsH sensor has the advantage of leaving the receptor more intact with respect to its ability to signal to G-proteins.

Further alternatives to assess GPCR activation are approaches based on protein complementation [185]. In this field enzyme fragment complementation (EFC) assays notably have the advantage that the signal is generated catalytically, and thus the assay can exhibit high sensitivity. Enzyme reporter proteins such as β-galactosidase, dihydrofolate reductase (DHFR) or lactamase have been utilized, all of which can turn over chromogenic or fluorogenic substrates. Luminescent signals can be generated, either with luciferase or β-galactosidase as an enzyme reporter. However, as split enzyme-based reporters require substrate incubation, these assays often need optimization with respect to concentration and incubation time to exclude the background signal caused by the substrate. Alternatively, complementation assays with split fluorescent proteins with direct read-outs, such as microscopy or scanning spectroscopy instruments, were developed for the detection of rapid interactions without interfering background signals [185].

#### Protein-protein interactions

An important aim in cell biology has been to identify and to observe dynamic interactions between protein molecules, as they execute the reactions of a particular biochemical pathway. Concerning GPCRs, despite elucidating ligand binding and signaling, the knowledge and investigation of conformational changes and further occurring protein-protein-interactions as well as their meaning for the life cycle of GPCRs and changes in cellular responses is of utmost interest. Frequently applied biochemical methods for the investigation of protein-protein-interactions are immunoprecipitation, photo-affinity labeling, cross-linking, size-separation chromatography and Western blot analysis. All these methods include cell lysis and are not able to follow protein-protein interaction in a living cell. The development of fluorophores and imaging techniques, such as FRET, BRET and BiFC, provide experimental alternatives to these denaturing techniques (e. g. immunoprecipitation). Fluorescence methods allow the localization of specific biomolecules in a time dependant manner, and accordingly also the protein-protein interaction processes. Accordingly, these techniques will help to find further answers to controversially discussed issues such as the role of interacting proteins in signal transduction cascades and their temporarily or permanent contacts.

Different FRET techniques and fusions with different spectral characteristics have been described and used for the study of protein interactions. Many modified versions of FRET were developed to suit individual needs, e.g. for large-scale quantitative analysis by flow cytometry [186,187]. FRET can be measured in different ways. The sensitized emission method is a spectroscopic approach in which the sample of interest is excited at the wavelength of the donor and thus the increase of the acceptor fluorescence is quantified. Applying this technique requires additional measurements of samples that only contain the donor or acceptor, respectively, for correct data evaluation. Acceptor photo-bleaching FRET using confocal microscopy has become widely employed and has the advantage to localize occurring FRET events at the subcellular level [188]. In this approach the reduced fluorescence intensity of the donor in the presence of the acceptor within a FRET is focused. To demonstrate interactions images before and after complete photo-bleaching of the acceptor are obtained. When the donor image is brighter following the acceptor photo-bleaching, then FRET can be assumed to have taken place. Within BRET approaches microscopy is rarely applied, the majority of studies utilize plate-reading instrumentation [189]. Besides the possibility to perform single cell BRET assays [190], there are many technical limitations because of the reduced intensity of light released when luciferase oxidizes its substrate. Thus the taking place, but not the locations of energy transfer can be investigated. However, advantages of the BRET technique include the independence of a light source to initiate the energy transfer and the lack of photo-bleaching. In contrast, the BiFC approach allows spectroscopic as well as microscopic examinations. The complementary parts of the different GFP variants, when brought in close vicinity, produce BiFC with unique spectral properties. Thus the technique allows detection of multiple as well as competing interactions *in vivo*. By combining BiFC with either FRET or BRET, it is theoretically possible to demonstrate the simultaneous interaction of three or more interacting partners. Recently, it was demonstrated that *Renilla *luciferase (RLuc) [191,192] can be used as a split enzyme. Accordingly BRET experiments also might be possible now to study the assembly of multiple proteins into a complex.

Comparing the three methods, which one is the most powerful? In fact, all three have advantages and disadvantages and specific applications they could be used for best. FRET and BRET are powerful techniques for real time experiments, with reversible energy transfer but limited dynamic range and the requirement of a complex multiwave lengths analysis that is prone to artefacts. In contrast, BiFC studies are no real time experiments, very sensitive and easy to measure, but represent the end-point as the fluorscence formation is irreversible. Localisation of the interaction is best studied with BiFC, whereas multiprotein dynamics are possible for competitive (multicolour BiFC) and cooperative (BiFC/BRET) interactions.

Through the application of these fluorescence based methods it is now widely accepted that the formation of homo- and/or heterodimers or higher order complexes is a universal aspect of GPCR biology [193] and could have important functional roles, e.g. in receptor maturation, function and trafficking [194,195]. A lot of GPCR systems have already been examined by these non-invasive methods [196] and these investigations have supported our understanding of the functional significance of homo- and heterodimerization of GPCRs [197].

Despite the wide-spread application of GFP and its variants, the recent development of self-labeling tags provides further alternatives of donor and acceptor receptor fusions within FRET experiments. Recently the SNAP™-tag technology was successfully applied in time-resolved FRET measurements [198]. The acyl carrier protein (ACP) labeling technique was used to simultaneously label the N-terminus of the neurokinin-1 receptor (NK1R) with Cy3 and Cy5 at different, but well-defined ratios and thus allowed FRET studies with high signal-to-noise ratios [199]. Also fluorescent antibodies were successfully used to detect receptor oligomerization. The various existing epitopes allow not only co-expression and co-visualization of two differently tagged GPCRs in one cell, but also provide information about possible protein-protein-interactions. Because of the existence of different fluorescent dye pairs, e.g. fluorescein isothiocyanate (FITC, donor) and rhodamine (acceptor), which have an overlap in the donor emission and the acceptor excitation spectra, labeled antibodies have already been applied in FRET studies to investigate GPCR oligomerization [200]. Fluorescent ligands are also used to study receptor subtype oligomerization by FRET. GPCR-dimers as GFP-fusions can be determined even when they still accumulate in the endosomal compartments. As receptor oligomerization is discussed in the context of receptor trafficking this is of major interest [201].

Receptor-mediated activation of a G-protein is an early event in signal transduction and is thought to be a result of a transient interaction between an agonist occupied receptor and its G-protein. However, also data from FRET and BRET studies provided evidence that GPCRs and G-proteins can form stable complexes [202,203]. G-protein β and γ subunits bind to each other with high affinity, forming a heterodimeric complex, which is essential for the stability of these peptides. The stability of the heterotrimeric complex formed by the association of G_α _and G_βγ _is a controversial. The hypothesis that G_β _dissociates from G_βγ _when heterotrimeric G-proteins are activated *in vivo *is generally accepted, however some experiments suggest that they do not. Both hypotheses have been based largely on data from *in vitro *experiments [203] and therefore FRET has been used to probe this interaction during signal transduction *in vivo*. However, the construction of a functional fluorescently tagged G_α _subunit was not as simple as producing their tagged and functional counterparts. Although functional G_α _fusion proteins have been prepared by inserting GFP variants into their α-helical domains [204,205], FRET experiments in intact cells also led to controversial results [177,178]. A decrease in FRET signal can be due to subunit dissociation, however a conformational change within the heterotrimeric complex can also produce the same result. The interpretation of changes in FRET can be even more complicated, assuming that each receptor monomer forms a complex with a G-protein. The resulting proximity of G-proteins could produce FRET between subunits of different G-proteins as well as between subunits within the same G-protein and changes in FRET may be due to conformational changes within an individual G-protein, between different G-proteins or a combination of both.

Prolonged agonist activation of a GPCR is followed by desensitization that occurs when G-protein coupled receptor kinases (GRKs) phosphorylate the agonist-occupied receptor and prevent further stimulation. This process facilitates the interaction of the receptor with the protein arrestin, resulting in internalization of the desensitized GPCR, further leading either to receptor down regulation or to resensitization and receptor recycling to the plasma membrane [206]. BRET is widely used to detect the interaction of arrestin with a GPCR [207-209] and FRET is applied to follow the time course of interaction [173,210]. BRET studies additionally probing the interaction between GPCRs and GRKs revealed that the time course for the interaction with arrestin lagged behind the interaction with GRK, which is consistent with the requirement, that GRK-catalyzed phosphorylation must precede arrestin binding [211].

## Conclusion

It is proven that fluorescence techniques are powerful tools for investigation of the very dynamic family of GPCRs to understand their subcellular localisation and to further elucidate key elements in GPCR trafficking and interaction with other signal pathways.

However, to obtain physiologically relevant results, some considerations have to be made. First, it is of utmost importance that the investigated GPCR, ligand or interacting protein is not influenced in its functionality by the fluorescent modification. Therefore careful characterizations are needed and to exclude interferences it might be helpful to apply different labels at different sites of the protein for data evaluation [212]. Additionally, the label should be as small as possible, the recent and ongoing development and optimization of self-labeling tags will be advantageous in this field. Using unnatural amino acid mutagenesis the site-specific incorporation of reactive keto groups, such as *p*-benzoyl-L-phenylalanine (Bzp) or *p*-acetyl-L-phenylalanine (Acp), into functional GPCRs and their ability to react with a variety of spectroscopic and other probes was previously described [213]. Because of their excellent fluorescent properties quantum dots are very attractive for labeling, however the full potential of QDs for cellular imaging has not yet been realized because of problems with large QD size, QD multivalency and the difficulty of delivering QDs into the cytosol. Recently, monovalent and reduced-size quantum dots were generated and successfully applied for receptor imaging in living cells [214].

Fluorescent antibodies provide a powerful tool for examining the cellular distribution of GPCRs. However, quantification is highly depended on the accessibility of – in most cases – the small epitope by the large antibody. The challenge is to develop even more high-affinity fluorophore- or enzyme-conjugated primary antibodies for one-step labeling assays on living cells. The generation of bright and stable dyes as well as pH sensitive ones, such as CypHer 5 [34], will lead to further insights into the life of GPCRs and will enable high-throughput screening applications. A new group of molecules, called affibody molecules, is especially interesting for imaging applications because of their small size (7–15 kDa) compared to antibodies. These proteins are composed of a three-helix bundle of 58 amino acids and are derived from the scaffold of one of the IgG-binding domains of the staphylococcal protein A [215]. The binding site is equivalent to an antibody with respect to the surface area. The size, the simple structure, the specific target recognition, the ease of production and the high stability give affibody molecules significant advantages over antibodies. These molecules can be labeled with fluorophores but also with radionuclides which make them promising candidates for GPCRs associated tumor diagnosis and therapy [216].

Recombinant DNA technologies have highly advanced fluorescence labeling as well as transfection and transgenic techniques that enable simple DNA delivery to cells that results in covalent labeling by using the protein expression system of the cell. However, expression levels in cell cultures may significantly differ from those in natural systems. Concerning the signaling and trafficking behavior of GPCRs the relationship between the occupation of the receptor by physiological levels of agonists and the initiation of translocation is an important issue. The general use of very high concentrations of agonist leaves open the possibility that the investigated processes are more pharmacological than physiological.

A major criticism of FRET/BRET studies used to investigate protein-protein-interactions, is that the required protein overexpression can result in RET attributed to a high incidence of random collisions, rather than direct protein-protein-interactions. If low expression levels can not be obtained by varying DNA amounts within transient cell transfections, stable cell transfections will provide an alternative, since there is a homogenous population of cells expressing the protein of interest at the same level. Another possibility is the baculovirus expression system which enables protein expression levels to be controlled more closely than with transient transfection, because protein expression can be titrated by adjusting the multiplicity of viral infection [217].

Since protein co-localization is the first prerequisite for interactions, this should be proven by fluorescence microscopy, and by using parallel labeling strategies to locate subcellularly the interaction of interest. For correct evaluation of FRET and BRET data appropriate controls have to be used to demonstrate the specificity of the interactions and to establish levels of RET considered to be background in any given experiment. The additional application of a biochemical approach might support the results. To validate the physiological role of the detected interaction studies in other, more natural cell systems, e.g. cell lines endogenously expressing one protein of interest, as well as investigations on tissues and animals will be indispensable in proving the relevance of the interactions in the future. For example, the *in vivo *co-expression of GPCRs has to be demonstrated in the same tissue, and ideally in the same cell for establishing the physiological relevance of receptor oligomerization. Functional cross-talk between the receptor signaling pathways as well as novel pharmacological and/or functional properties will provide evidence for the mechanism by which receptor-receptor-interactions modulate cellular activity [218].

An exciting application of GPCR-GFP chimeras involves their use in genetic screens in genetically tractable organisms such as yeast, e.g. to identify mutant yeast strains in which the receptor is mis-localized. Such strategies contribute greatly to the identification of new components involved in GPCR targeting and trafficking in additional model organisms [219]. New approaches using whole organisms, in which the GFP-chimera can be expressed under the control of the endogenous promoter, e.g. invertebrates as *C. elegans *or mouse models, allow cell biological, molecular and biochemical results to be interpreted in a physiologically relevant context and to be compared to those observed in cultured cells [220,221]. GFP and its variants as reporters represent the next step in mouse genome engineering technology by opening up the possibility of combinatorial non-invasive reporter usage within a single animal, e.g. for gene-expression, as well as for co-visualization and FRET assays [222].

In summary, many issues concerning the life of GPCRs can be addressed by fluorescence techniques, however many remain challenging. Further rapid advances in labeling and imaging technology can be expected and their parallel as well as their combined application will provide novel insights that will also broaden the range of new therapeutic interventions.

## Competing interests

The authors declare that they have no competing interests.

## Authors' contributions

IB wrote the manuscript and prepared the figures and tables. AGBS supervised the process of writing and has critically revised the manuscript. Both authors read and approved the final manuscript.
